# IGF-1 Attenuates Hypoxia-Induced Atrophy but Inhibits Myoglobin Expression in C2C12 Skeletal Muscle Myotubes

**DOI:** 10.3390/ijms18091889

**Published:** 2017-09-01

**Authors:** Eva L. Peters, Sandra M. van der Linde, Ilse S. P. Vogel, Mohammad Haroon, Carla Offringa, Gerard M. J. de Wit, Pieter Koolwijk, Willem J. van der Laarse, Richard T. Jaspers

**Affiliations:** 1 Laboratory for Myology, Faculty of Behavioral and Movement Sciences, Department of Human Movement Sciences, Amsterdam Movement Sciences, Vrije Universiteit Amsterdam, De Boelelaan 1108, 1081 HZ Amsterdam, The Netherlands; el.peters@vumc.nl (E.L.P.); desandra@hotmail.com (S.M.v.d.L.); ilsevogel@gmail.com (I.S.P.V.); m.h.mohammadazam@vu.nl (M.H.); c.offringa@vu.nl (C.O.); g.m.j.de.wit@vu.nl (G.M.J.d.W.); 2 Department of Physiology, Amsterdam Cardiovascular Sciences, VU University Medical Center, De Boelelaan 1108, 1081 HZ Amsterdam, The Netherlands; p.koolwijk@vumc.nl (P.K.); wj.vanderlaarse@vumc.nl (W.J.v.d.L.)

**Keywords:** hypoxia, myoglobin, hypertrophy, anabolic signaling, C2C12, fatty acid, mTOR, mitochondrial biosynthesis, succinate dehydrogenase, myogenic regulatory factors, VEGF

## Abstract

Chronic hypoxia is associated with muscle wasting and decreased oxidative capacity. By contrast, training under hypoxia may enhance hypertrophy and increase oxidative capacity as well as oxygen transport to the mitochondria, by increasing myoglobin (Mb) expression. The latter may be a feasible strategy to prevent atrophy under hypoxia and enhance an eventual hypertrophic response to anabolic stimulation. Mb expression may be further enhanced by lipid supplementation. We investigated individual and combined effects of hypoxia, insulin-like growth factor (IGF)-1 and lipids, in mouse skeletal muscle C2C12 myotubes. Differentiated C2C12 myotubes were cultured for 24 h under 20%, 5% and 2% oxygen with or without IGF-1 and/or lipid treatment. In culture under 20% oxygen, IGF-1 induced 51% hypertrophy. Hypertrophy was only 32% under 5% and abrogated under 2% oxygen. This was not explained by changes in expression of genes involved in contractile protein synthesis or degradation, suggesting a reduced rate of translation rather than of transcription. Myoglobin mRNA expression increased by 75% under 5% O_2_ but decreased by 50% upon IGF-1 treatment under 20% O_2_, compared to control. Inhibition of mammalian target of rapamycin (mTOR) activation using rapamycin restored Mb mRNA expression to control levels. Lipid supplementation had no effect on Mb gene expression. Thus, IGF-1-induced anabolic signaling can be a strategy to improve muscle size under mild hypoxia, but lowers Mb gene expression.

## 1. Introduction

Chronic diseases and aging are conditions associated with a loss in muscle mass and increased fatigability [1]. One of the contributing factors to the deterioration of skeletal muscle may be hypoxia and the chronic disease associated effects resemble those that have been reported in humans after experimental exposure to chronic hypoxia [2,3,4,5]. Humans show decreased muscle fiber cross-sectional area, and constant or lower mitochondrial volume, the latter being indicative for a lower oxidative capacity [3,6,7]. Similar effects were observed in C2C12 mouse skeletal myotubes, engineered skeletal muscle tissue, and in rodents where exposure to hypoxia also reduced time to fatigue during treadmill running [8,9,10,11,12,13,14,15].

One potent way to increase muscle strength is resistance exercise. However, studies on resistance exercise under hypoxia showed contradictory effects. Whereas some showed a blunted hypertrophic response to training under hypoxia [12,16,17], others suggested that training under hypoxia may prevent atrophy and can even enhance hypertrophy and oxidative metabolism [2,18,19,20,21,22,23,24]. Insulin-like growth factor (IGF)-1 is well known for its anabolic effects by activation of mammalian target of rapamycin (mTOR) and its downstream effector p70S6K [25], although it is currently unknown whether IGF-1 treatment can oppose hypoxia-induced skeletal muscle atrophy [26,27,28].

With hypertrophy, the diffusion distance for oxygen to the core of the cell increases and thereby imposes a size constraint on the muscle fiber [29,30,31]. Myoglobin (Mb) facilitates oxygen diffusion within the cell and serves as an oxygen buffer [32,33]. In hypertrophied muscle fibers and cardiac myocytes working at maximal oxygen uptake (VO_2_max), increased intracellular oxygen transport via Mb is required to prevent hypoxic cell cores [30,34]. Therefore, inadequate oxygen supply or hypoxia limits hypertrophy. Increasing Mb expression may thus serve as a strategy to prevent atrophy under hypoxia and enhance an eventual hypertrophic response to IGF-1 [5].

When hypoxia is combined with exercise, Ca^2+^ activates calcineurin (CN) [35]. CN then dephosphorylates myocyte enhancer factor 2 (MEF2) and nuclear factor of activated T-cells (NFAT) to induce translocation of these transcription factors to the nucleus, and subsequent Mb gene transcription [36]. Indeed, the combination of hypoxia and exercise increased Mb expression in different animal models as well as in human [5,35,37,38]. Activation of the Akt-mTOR pathway causes hyperphosphorylation of NFAT in C2C12 myotubes, which prevented nuclear entry of NFAT and blunted NFAT-c1 activation upon calcium ionophore treatment [26]. It is unclear, however, whether IGF-1-induced mTOR activation also prevents NFAT-induced Mb transcription.

A possible way to enhance Mb expression in a CN-NFAT independent manner could be lipid supplementation [39]. Indeed, supplementation of poly-unsaturated fatty acids (PUFAs) in patients with chronic obstructive pulmonary disease (COPD) and healthy rats showed marked improvements in exercise capacity [40,41,42,43]. In addition, lipid supplementation previously showed effects on genes involved in protein breakdown and mitochondrial biogenesis [44,45]. However, no experimental studies were undertaken to investigate whether increased Mb expression via a CN-NFAT independent pathway, combined with a hypertrophic stimulus, has synergistic effects on muscle hypertrophy and oxidative capacity under hypoxia.

The aims of this study were therefore to investigate whether: (1) IGF-1 can attenuate hypoxia-induced atrophy; (2) IGF-1 inhibits Mb gene expression by hyperphosphorylation of NFAT; and (3) increased Mb expression via a CN-NFAT independent pathway combined with IGF-1 treatment and hypoxia has synergistic effects on myotube hypertrophy and its regulation.

We hypothesized that IGF-1 antagonizes the atrophic effects of hypoxia in C2C12 myotubes but inhibits Mb gene transcription via mTOR-induced hyperphosphorylation of NFAT. Further, we expect that a CN-NFAT independent increase in Mb, induced by lipid supplementation, enhances the hypertrophic effects of IGF-1.

## 2. Results

### 2.1. Effects of Hypoxia, IGF-1 and Lipids on Myotube Size

**Figure 1:**
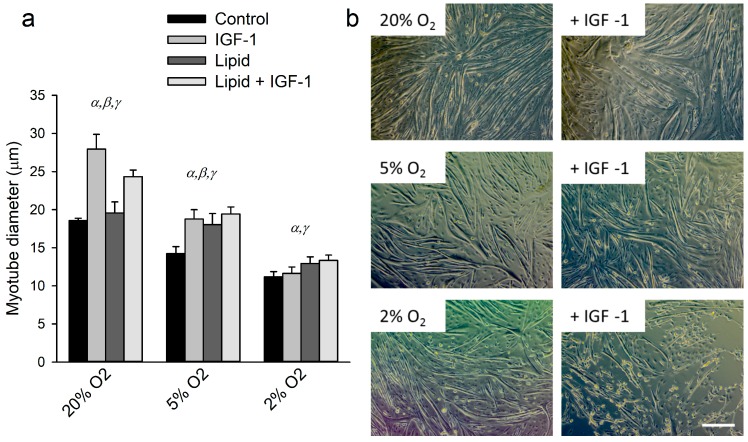
Insulin-like growth factor (IGF)-1-induced hypertrophy is abrogated under hypoxia: (**a**) fiber diameter of myotubes cultured the last 24 h of differentiation under different oxygen tensions, with and without supplementation of IGF-1 and lipid; and (**b**) representative photographs of control- and IGF-1 supplemented cells in all three oxygen tensions. α: significant effect of oxygen tension compared to other O_2_ tensions; β: significant effect of IGF-1 treatment within that specific oxygen tension; γ: significant overall effect of lipid supplementation. Values are given as mean ± SEM, *n* = 6. Scale bar represents 250 µm.

Culturing for 24 h under hypoxia caused a decrease in mean myotube diameter (*p* < 0.01; Figure 1a,b) by 24% under 5% O_2_ and by 40% under 2% O_2_ compared to 20% O_2_. Under 20% O_2_, supplementation of IGF-1 increased myotube diameter by 51%. This increase was 32% under 5% O_2_ and hypertrophy was absent under 2% O_2_, indicating that at lower oxygen tensions the hypertrophic response was attenuated and eventually blunted. Lipid supplementation had an overall hypertrophic effect (*p* = 0.04) but did not enhance IGF-1-induced hypertrophy.

### 2.2. Effects of Hypoxia, IGF-1 and Lipids on Regulators of Protein Synthesis and Degradation

**Figure 2:**
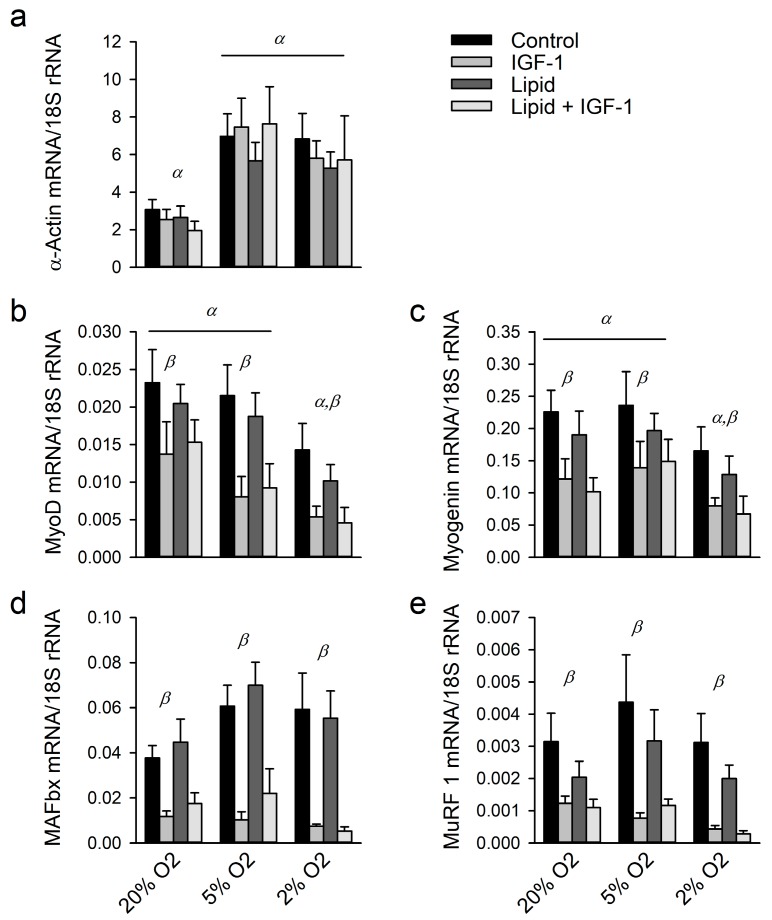
Regulators of protein synthesis are decreased under hypoxia and following IGF-1 supplementation, whereas regulators of protein synthesis are only slightly increased under hypoxia: (**a**) mRNA expression levels of α-actin under different oxygen tensions, with and without 24 h supplementation of IGF-1 and lipid; (**b**,**c**) mRNA expression levels of differentiation markers MyoD and myogenin; and (**d**,**e**) mRNA expression levels of protein degradation markers Muscle Atrophy F-box (MAFbx) and Muscle RING finger 1 (MuRF1). Please note that the interaction for IGF-1 treatment with oxygen tension is not depicted. α: significant effect of oxygen tension compared to other O_2_ tensions denoted with α; β: significant overall effect of IGF-1 treatment; γ: significant overall effect of lipid supplementation. Although β and γ designate overall effects, symbols are placed above each oxygen tension for clarity. Values are given as mean ± SEM, *n* = 6.

Next, we looked further into the underlying mechanisms of hypoxia-induced atrophy and the blunted hypertrophic response to IGF-1. Surprisingly, α-actin mRNA expression levels increased in hypoxia (*p* < 0.01; Figure 2a), whereas IGF-1 had no effect on α-actin mRNA expression (*p* = 0.37). To explain this increase in α-actin expression, we investigated mRNA expression levels of MyoD and myogenin, both involved in the regulation of contractile protein gene expression and activation of satellite cells. Both genes had lower expression levels following IGF-1 treatment in all three oxygen conditions (*p* < 0.01 for both; Figure 2b,c). In addition, oxygen had a significant main effect on MyoD and myogenin expression levels (*p* < 0.01 for both) with expression levels of both being lower under 2% O_2_ than those under 20% (*p* < 0.01 and *p* < 0.05, respectively) and under 5% oxygen (*p* < 0.05 and *p* < 0.01, respectively).

Muscle RING finger 1 (MuRF1) and Muscle atrophy F-box (MAFbx) expression levels decreased following IGF-1 treatment under all three oxygen conditions (*p* < 0.01 and *p* < 0.05, respectively; Figure 2d,e), while neither MuRF1 nor MAFbx expression levels were changed by lipid supplementation (*p* = 0.24 and *p* = 0.64 respectively). The interaction effect between IGF-1 supplementation and oxygen conditions (*p* < 0.01) revealed that, in absence of IGF-1, MAFbx mRNA expression levels increased under 5% compared to those under 20% O_2_, whereas they decreased under 2% O_2_ when IGF-1 was added. In presence of IGF-1, MuRF1 mRNA expression levels under 5% and 2% O_2_ were lower than those under 20% O_2_ whereas this was not the case in the absence of IGF-1. These results suggest that protein degradation is at most slightly increased under hypoxia and strongly decreased upon IGF-1 supplementation.

### 2.3. Effects of Hypoxia, IGF-1 and Lipids on Regulators of Metabolism

**Figure 3:**
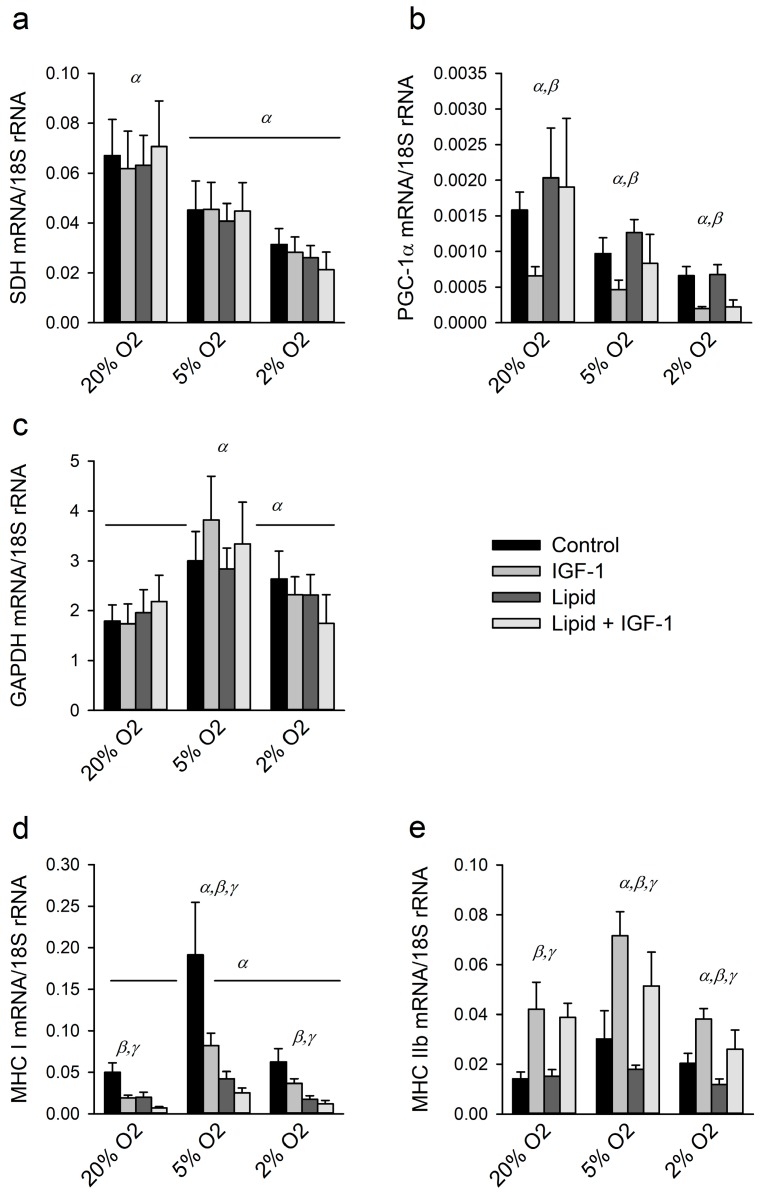
Hypoxia likely causes a reduction in oxidative metabolism and a shift to more glycolytic metabolism: (**a**,**b**) mRNA expression levels of SDH and PGC-1α for myotubes cultured the last 24 h of differentiation under different oxygen tensions, with and without IGF-1 and lipid as markers of oxidative metabolism; (**c**) mRNA expression levels of GAPDH was measured as marker of glycolytic metabolism; and (**d**,**e**) mRNA expression levels of myosin heavy chain (MHC) I and -IIB were measured as markers for fiber type switching. α: significant effect of oxygen tension compared to other O_2_ tensions denoted with α; β: significant overall effect of IGF-1 treatment; γ: significant overall effect of lipid supplementation. Although β and γ designate overall effects, symbols are placed above each oxygen tension for clarity. Values are given as mean ± SEM, *n* = 6.

Figure 3 shows mRNA expression levels of genes related to oxidative- or glycolytic metabolism and myosin heavy chain types. Oxidative enzyme capacity as reflected by succinate dehydrogenase (SDH) mRNA expression was decreased under hypoxia in a dose dependent manner (*p* < 0.001; Figure 3a), whereas neither lipid supplementation (*p* = 0.51) nor IGF-1 treatment (*p* = 0.50) affected SDH mRNA expression levels.

Peroxisome proliferator-activated receptor-γ coactivator (PGC)-1α was also negatively affected by hypoxia (*p* < 0.001; Figure 3b). IGF-1 treatment caused a decrease in PGC-1α mRNA expression levels in all three oxygen conditions (*p* < 0.001), whereas lipid supplementation had no effect on PGC-1α mRNA expression levels (*p* = 0.26).

Glyceraldehyde-3-phosphate dehydrogenase (GAPDH) was measured as a marker of glycolytic metabolism and was increased by lowering oxygen concentrations to 5% only (*p* < 0.001; Figure 3c). Neither IGF-1 nor lipid supplementation altered GAPDH mRNA expression levels (*p* = 0.73 and *p* = 0.53, respectively).

We further investigated whether these metabolic alterations coincided with myosin heavy chain (MHC) type switching. Compared to 20% O_2_, mRNA expression levels of slow MHC type I increased almost four-fold under 5% O_2_ (*p* < 0.001; Figure 3d) but decreased upon IGF-1 treatment and lipid supplementation (*p* < 0.001 for both). By contrast, mRNA expression levels of the fast MHC type IIB were increased by IGF-1 treatment (*p* < 0.001; Figure 3e). Relative to 20% O_2_ MHC IIB mRNA expression levels were higher under 5% O_2_ than under 2% O_2_ (*p* < 0.01). In addition, lipid supplementation lowered MHC IIB expression (*p* < 0.05). These results indicate that lowering oxygen levels caused a reduction in oxidative metabolism and that IGF-1 likely favored a shift towards glycolytic metabolism and expression of the fast MHC type IIB.

### 2.4. Effects of Hypoxia, IGF-1 and Lipids on Markers of Oxygen Transport

**Figure 4:**
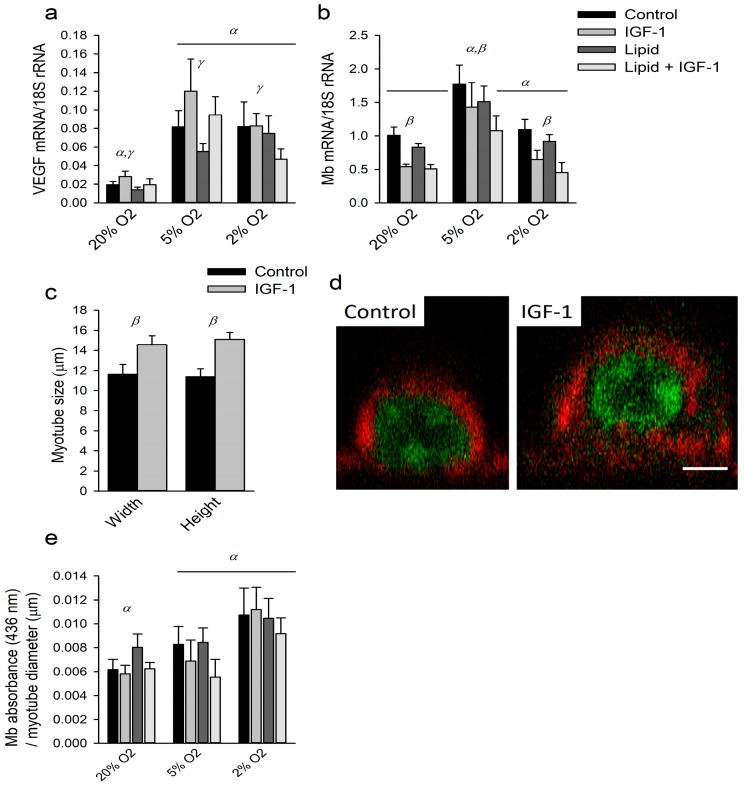
IGF-1 lowers myoglobin (Mb) mRNA expression but not Mb protein concentration in the cells: (**a**,**b**) mRNA expression levels of vascular endothelial growth factor (VEGF) and Mb for myotubes cultured the last 24 h of differentiation under different oxygen tensions, with and without IGF-1 and lipid; (**c**) in a separate experiment, height and width of myotubes was measured (*n* = 20 myotubes); (**d**) representative examples of cross-sections of individual myocytes live stained in red for F-actin and in green for DNA (scale bar represents 5 μm); and (**e**) absorbance due to Mb peroxidase activity normalized by myotube diameter as a measure of concentration. α: significant effect of oxygen tension compared to other O_2_ tensions denoted with α; β significant overall effect of IGF-1 treatment; γ: significant overall effect of lipid supplementation. Although β and γ designate an overall effect, for clarity symbols are placed above every oxygen tension. Values are given as mean ± SEM, *n* = 6.

To investigate the effects of hypoxia, IGF-1 treatment and lipid supplementation on the regulation of oxygen supply to the cells, we investigated mRNA expression levels of vascular endothelial growth factor (VEGF) and Mb, as well as Mb protein expression as is shown in Figure 4. VEGF expression levels increased under hypoxia (*p* < 0.001; Figure 4a). IGF-1 treatment did not show any effect on VEGF expression levels (*p* = 0.19), whereas lipid supplementation decreased VEGF expression levels (*p* < 0.001).

Oxygen tension had a significant main effect on Mb mRNA expression levels (*p* < 0.001; Figure 4b) which were increased under 5% O_2_ compared to 20% (*p* < 0.001). However, under 2% O_2_, expression levels of Mb mRNA did not significantly differ from those in 20% O_2_ (*p* = 1.00). Furthermore, upon IGF-1 treatment, Mb mRNA expression levels decreased in all three oxygen conditions (*p* < 0.001). Lipid supplementation itself did not significantly increase Mb mRNA expression levels (*p* = 0.1) and the absence of any significant interaction with IGF-1 indicates that lipid supplementation also could not prevent the IGF-1-induced decrease in Mb mRNA expression.

The Mb content was measured as absorbance using a calibrated histochemical method based on peroxidase activity [46]. Since absorbance measurements depend on the path length, we verified whether IGF-1-induced hypertrophy occurs equally in all radial directions. The increase in width did not differ from the increase in height (*p* = 0.34). On average, upon IGF-1 treatment myotube width and height increased by 25% and 33%, respectively (*p* < 0.001 for both; Figure 4c,d). This suggests that IGF-1 induced myotube hypertrophy occurred uniformly in radial directions. Since absorbance due to Mb peroxidase activity is proportional to path length up to at least 16 μm [46], Mb absorbance normalized by myotube diameter is taken as a measure of Mb concentration because Lambert-Beer’s law applies.

Despite an increase in Mb mRNA expression levels in 5% O_2_, Mb content per myotube did not change in any of the oxygen conditions, nor due to IGF-1 treatment or lipid supplementation (results not shown, *p* = 0.33, *p* = 0.35 and *p* = 0.38, respectively). However, Mb concentration was higher under 2% O_2_ compared to 5% and 20% O_2_ (*p* < 0.01), whereas IGF-1 and lipid supplementation showed no effects (*p* = 0.19 and *p* = 0.93, respectively, Figure 4e).

### 2.5. IGF-1 Inhibits Myoglobin mRNA Expression via mTOR Activation

**Figure 5:**
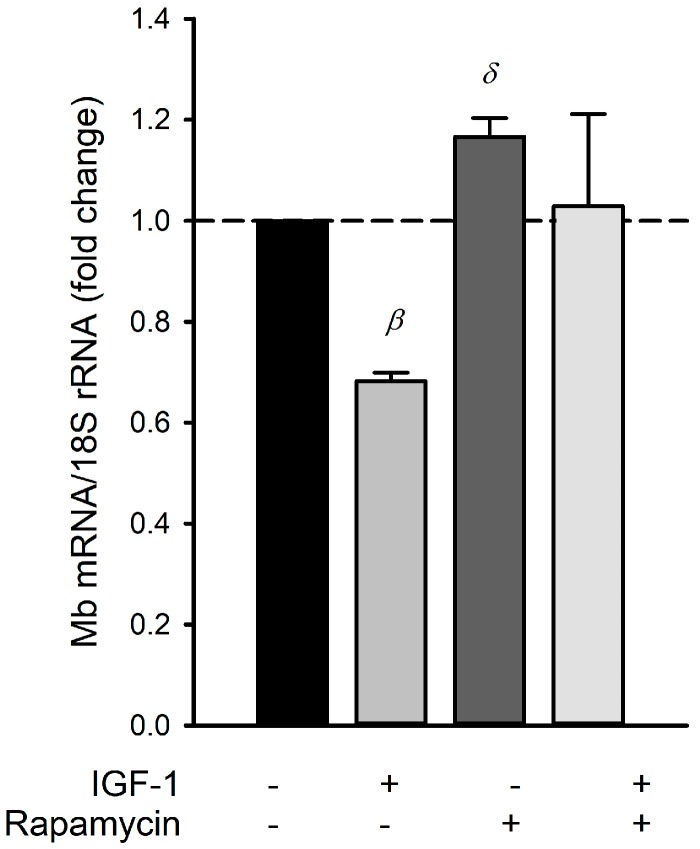
IGF-1 inhibits Mb mRNA expression, which can be restored by inhibition of mammalian target of rapamycin (mTOR). Mb mRNA expression levels relative to control for myotubes treated for 24 h with IGF-1, myotubes treated with rapamycin or a combination. Myotubes were cultured under 20% O_2_. β: significant effect of IGF-1 treatment; δ: significant effect of rapamycin treatment. Values are given as mean ± SEM, *n* = 4.

To investigate whether the inhibition of Mb expression by IGF-1 was indeed caused by hyperphosphorylation of NFAT via mTOR, we inhibited IGF-1-induced mTOR signaling by rapamycin. Mb mRNA expression levels relative to control levels are shown in Figure 5. Both IGF-1 and rapamycin showed a significant main effect on Mb mRNA expression levels (*p* < 0.05 for both). An interaction for IGF-1 x rapamycin (*p* < 0.05) was present, indicative of the opposite effects of both treatments.

## 3. Discussion

As expected, myotube diameter decreased under hypoxia, as well as mRNA expression levels of SDH and its regulator PGC-1α. Surprisingly, the decrease in diameter was accompanied by increased, rather than decreased, α-actin mRNA expression levels. MyoD and myogenin are myogenic factors and mRNA expression of myogenin is related to α-actin promotor activity [47]. We therefore determined mRNA expression levels of MyoD and myogenin to investigate regulation of protein synthesis at the transcriptional level, and showed a decrease under 2% but not under 5% O_2_, consistent with the literature [48,49,50]. On the other hand, MAFbx mRNA expression levels increased only under 5% O_2_, whereas MuRF1 mRNA expression levels remained unaltered, suggesting only a minor -if any- increase in protein degradation. Thus, mRNA expression levels of our markers for protein synthesis and degradation cannot explain why α-actin mRNA expression levels were increased concomitant with the decrease in myotube diameter.

Alternatively, mRNA translation may be impaired under hypoxia. In vivo, in both rat and human, contradictory results regarding the phosphorylation of Akt, mTOR and p70S6K have been reported [12,51,52,53]. However, C2C12 myoblasts cultured under hypoxia showed reduced basal Akt-mTOR activation, as well as blunted IGF-1-induced Akt-mTOR activation [50]. In addition, C2C12 myotubes cultured under 2% O_2_ for 48 h showed lower IGF-1-induced phosphorylation of Akt, mTOR and p70S6K after 60 and 180 min of IGF-1 stimulation compared to normoxic controls. Furthermore, in engineered skeletal muscle constructs a reduction in p70S6K phosphorylation following 24 h culture under 1% O_2_ was shown recently [15]. These results indicate that the rate of translation is indeed inhibited by hypoxia [53,54].

### 3.1. IGF-1-Induced Hypertrophy Is Limited by Hypoxia

We investigated whether IGF-1 can prevent hypoxia-induced atrophy. Although IGF-1 induced hypertrophy in myotubes cultured under 20% and 5% O_2_, the amount of hypertrophy under 5% O_2_ was 80% of that under 20% O_2_ and thereby only opposed the hypoxia-induced atrophy. In culture under 2% O_2_, atrophy was not prevented by IGF-1.

IGF-1 treatment markedly decreased MuRF1 and MAFbx mRNA expression levels under all three oxygen conditions, indicating that protein degradation was indeed decreased. Surprisingly, MyoD and myogenin mRNA expression levels also decreased following IGF-1 treatment, whereas α-actin mRNA expression levels remained unaffected by IGF-1. Although generally associated with myogenesis [55,56], doses of IGF-1 similar to the present dose (13 nM) previously diminished myogenic protein expression in C2C12 myoblasts to almost undetectable levels 24 h after plating from suspension whereas expression increased above control 72 h after plating, also dependent on cell density [57]. Thus, the response of MyoD and myogenin to IGF-1 treatment may depend on timing and duration of the treatment and/or on local PO_2_. Future investigations should reveal whether prolonged anabolic signaling in hypoxia can eventually induce hypertrophy.

### 3.2. IGF-1 Inhibits Myoglobin Gene Expression via Activation of mTOR

In C2C12 myotubes, IGF-1 has been shown to cause hyperphosphorylation of NFAT-c1 by mTOR and thereby IGF-1 prevented calcium ionophore-induced activation of NFAT-c1 [26]. Here, we show that IGF-1 treatment also lowers Mb mRNA expression levels. This is likely due to the hyperphosphorylation of NFAT by mTOR, since inhibition of mTOR by rapamycin increased Mb mRNA expression levels.

Despite decreased Mb mRNA expression levels following IGF-1 treatment, the Mb absorbance due to peroxidase activity was similar for all conditions. However, myotube diameter decreased under hypoxia. Assuming that myotube atrophy also occurred uniformly in all radial directions, we conclude that the concentration of Mb was increased under 2% O_2_, as reflected by a higher Mb absorbance/diameter ratio. Thus, despite an increase in Mb mRNA expression under hypoxia, the total content of Mb per unit of myotube length did not increase and the increase in concentration was only due to myotube atrophy.

### 3.3. Lipid Supplementation Does Not Increase Myoglobin Gene Expression

We hypothesized that Mb expression can be increased by lipid supplementation [39]. We found that lipid supplementation had no effect on either Mb mRNA or protein expression. Differences in timing and duration of the exposure may explain the discrepancy between present and previous results [39,58]. Particularly in the study by Schlater et al. myotubes were cultured with lipid during seven days of differentiation, whereas, in our study, lipids were present only for the last 24 h of differentiation. Possibly, lipid supplementation primes skeletal muscle to enhance Mb expression, which would agree with previous findings in primary myotubes from Weddel seal [58]. In rats fed high-fat diets for 32 weeks, starting at six weeks of age, a positive effect on Mb expression was also observed indicating that lipid supplementation exerts its effect on Mb expression not only during myogenesis. It may be that the longer duration of supplementation explains the difference in results of the present study compared to previous research [39,58]. We conclude that one day of supplementation is not sufficient. However, lipid supplementation reduced VEGF mRNA expression. Since VEGF may induce Mb expression [59], lipid supplementation can have inhibited, rather than stimulated Mb expression via VEGF signaling.

It should be noted that some studies used albumin as a carrier for the uptake of fatty acids, which could also explain the absence of lipid-induced Mb expression [44,60]. However, the increase in Mb expression previously reported by Schlater et al. was found without addition of albumin to the culture medium. In addition, we show an effect of lipid supplementation on myotube diameter and mRNA expression levels of VEGF and both MHC types, indicating that supplementation affected gene expression. It remains elusive whether lipid supplementation can induce fiber type switching and explain the effects of lipid supplementation in vivo.

We conclude that the hypertrophic response to IGF-1 is blunted under hypoxia. Although IGF-1 can attenuate hypoxia-induced atrophy of myotubes to a limited extent, it inhibits Mb mRNA expression. This cannot be circumvented following 24 h of lipid supplementation.

## 4. Materials and Methods

### 4.1. Cell Culture and Myotube Analyses

C2C12 myotubes were grown for four days in Dulbecco’s Modified Eagle’s Media (DMEM, Life Technologies, Carlsbad, CA, USA), supplemented with 10% fetal bovine serum (FBS, Thermo Scientific, Waltham, MA, USA), 1% Penicillin/Streptomycin (PS, Life Technologies, Carlsbad, CA, USA), and 0.5% fungizone (Life Technologies, Carlsbad, CA, USA) and kept in a humidified incubator at 37 °C, 5% CO_2_. Differentiation was induced at 60–70% confluency by changing the media to DMEM, supplemented with 2% horse serum (HS, GE Healthcare Life Sciences, Little Chalfont, UK), 1% PS and 0.5% fungizone (Life Technologies, Carlsbad, CA, USA), for four days. After 3 days of differentiation, myotubes were cultured for 24 h under different oxygen tensions (20%, 5% or 2% O_2_) using a custom designed hypoxia workstation as described before [61]. Myotubes were cultured in either differentiation medium, or in medium supplemented with IGF-1 (100 ng/mL, Peprotech, Rocky Hill, NJ, USA), a mixture of lipids (5% lipid-supplemented media (2 μg/mL arachidonic acid, 10 μg/mL each of linoleic, linolenic, myristic, oleic, palmitic and stearic fatty acids), Sigma, St. Louis, MO, USA), or a combination thereof. Rapamycin (2 ng/mL, Bioaustralis, Smithfield NSW, Australia) was added one hour prior to IGF-1 treatment and in 20% oxygen conditions only.

Four photographs of each well were taken at 10× magnification after the 24 h treatment. Diameters were measured in 20 myotubes (5 in each image) at 5 equidistant locations along the length of the cell using ImageJ (http://rsbweb.nih.gov/ij/, National Institutes of Health, Bethesda, MD, USA) and taking into account the pixel-to-aspect ratio. All original data can be found in Supplementary Materials.

### 4.2. Live Cell Imaging

To assess whether IGF-1-induced hypertrophy was equal in all radial directions C2C12 myotubes were cultured in 8-wells Ibidi-treated plates (Ibidi) as described above and either or not treated with IGF-1 for 24 h. Myotubes were stained in red for F-actin filaments using Sir-Actin (0.62–2.5 μM, Cytoskeleton Inc., Denver, CO, USA) and in green for the nucleus using Syto-9 (200 nM, Thermo Scientific, Waltham, MA, USA). Both stains were incubated for 4 h. Verapamil (10 μM, Cytoskeleton, Inc., Denver, CO, USA) was added to prevent excretion of the staining by the myotubes. Images were captured using a SP8 STED microscope (Leica microsystems GmbH, Wetzlar, Germany). 3D reconstructions were created from Z-stacks with 0.3 μm spacing. Height and width of the cells were measured in about 20 myotubes in each condition using The Medical Imaging Interaction Toolkit (MITK, http://www.mitk.org, Heidelberg, Germany) at 5 equidistant locations along the length of the myotubes.

### 4.3. Myoglobin Concentration

After culture, while remaining adhered to culture discs, myotubes were fixated for 10 min using glutaraldehyde (Sigma, St. Louis, MO, USA) [62] and subsequently incubated for 60 min in a buffer with O-tolidine (Sigma, St. Louis, MO, USA) and T-butylhydroperoxide (Fluka Chemie GmbH, Buchs, Switzerland) [62]. Images were taken at 5× magnification (Leica DMRB, Wetzlar, Germany). Absorbance was measured at 436 nm. The method was validated using gelatin sections containing known concentrations of horse myoglobin [46]. For each condition 20 myotubes were measured as described above.

### 4.4. Quantitative Polymerase Chain Reaction (qPCR)

Cells were harvested for RNA isolation in TRIreagent (Life Technologies, Carlsbad, CA, USA) and stored at −80 °C. RNA was isolated using RiboPure^TM^kit (Applied Biosystems, Foster City, CA, USA) and converted to cDNA with high-capacity RNA to cDNA master mix (Applied Biosystems, Foster City, CA, USA). cDNA was diluted 10× and stored at −20 °C until further use. For each gene target 5 µL of cDNA was amplified in duplicate using Fast SYBR Green Mastermix (Applied Biosystems, Foster City, CA, USA) on a StepOne Real-Time PCR system (Applied Biosystems, Foster City, CA, USA). Primers are listed in Table 1. Mean cycle thresholds were converted to relative expressions by subtraction of the 18S rRNA cycle threshold and determination of 2^−Δ*C*t^ [63].

### 4.5. Statistical Analysis

Three-way analysis of variance (ANOVA) was performed with factors oxygen tension, IGF-1 and lipid supplementation. Normality of the data was checked using Shapiro–Wilk tests. In the case of non-normality, ANOVA was performed on logarithmic transformed data. Equality of variances was verified using Levene’s test. Significant main effects were further investigated using Bonferroni multiple comparisons. Significant interaction effects were followed-up by one-way ANOVA using Bonferroni correction. Values are given as mean ± standard error of the mean (SEM); *p* < 0.05 was considered statistically significant. Unless stated otherwise, *n* = 6.

## Figures and Tables

**Table 1:** Primers used for PCR analysis. GAPDH glyceraldehyde-3-phosphate dehydrogenase; MAFbx muscle atrophy F-box; Mb Myoglobin; MHC Myosin Heavy Chain; MuRF1 muscle RING finger 1; PGC-1α peroxisome proliferator-activated receptor-γ coactivator-1α; SDH succinate dehydrogenase; VEGF vascular endothelial growth factor.

Target mRNA	Forward	Reverse
18S	GTAACCCGTTGAACCCCATT	CCATCCAATCGGTAGTAGCG
GAPDH	TGAAGCAGGCATCTGAGGG	CGAAGGTGGAAGAGTGGGAG
MAFbx	AGACTGGACTTCTCGACTGC	TCAGCTCCAACAGCCTTACT
Mb	GGAAGTCCTCATCGGTCTGT	GCCCTTCATATCTTCCTCTGA
MHC I	AGATCCGAAAGCAACTGGAG	CTGCCTTGATCTGGTTGAAC
MHC IIB	CAACTGAGTGAAGTGAAGACC	AGCTGAGAAACCATAGCGTC
MuRF1	GGGCTACCTTCCTCTCAAGTGC	CGTCCAGAGCGTGTCTCACTC
MyoD	AGCACTACAGTGGCGACTCA	GCTCCACTATGCTGGACAGG
Myogenin	CCCAACCCAGGAGATCATTT	GTCTGGGAAGGCAACAGACA
PGC-1α	ACACAACCGCAGTCGCAACA	GGGAACCCTTGGGGTCATTTGG
SDH	GTCAGGAGCCAAAATGGCG	CGACAGGCCTGAACTGC
α-Actin	GGCCAGAGTCAGAGCAGCAGAAAC	CACCAGGCCAGAGCCGTTGT
VEGF	CTGTAACGATGAAGCCCTGGAGTG	GGTGAGGTTTGATCCGCATGATCT

## Supplementary Materials

Supplementary materials can be found at www.mdpi.com/1422-0067/18/9/1889/s1.

## Abbreviations

CN	Calcineurin
COPD	Chronic Obstructive Pulmonary Disease
DMEM	Dulbecco’s Modified Eagle’s Media
FBS	Fetal Bovine Serum
GAPDH	Glyceraldehyde-3-phosphate dehydrogenase
HS	Horse Serum
IGF-1	Insulin-like Growth Factor 1
Mafbx	Muscle atrophy F-box
Mb	Myoglobin
MEF2	Myocyte Enhancer Factor 2
MHC	Myosin Heavy Chain
MITK	Medical Imaging Interaction Toolkit
mTOR	Mammalian Target Of Rapamycin
MuRF1	Muscle RING finger 1
NFAT	Nuclear Factor of Activated T-cells
p70S6K	p70-S6 Kinase 1
PGC-1α	Peroxisome Proliferator-Activated Receptor-γ Coactivator 1α
PUFA	Poly-Unsaturated Fatty Acid
qPCR	Qualitative Polymerase Chain Reaction
SDH	Succinate Dehydrogenase
SEM	Standard Error of the Mean
SR	Sarcoplasmic Reticulum
VEGF	Vascular Endothelial Growth Factor
